# Absolute Position Sensing Based on a Robust Differential Capacitive Sensor with a Grounded Shield Window

**DOI:** 10.3390/s16050680

**Published:** 2016-05-11

**Authors:** Yang Bai, Yunfeng Lu, Pengcheng Hu, Gang Wang, Jinxin Xu, Tao Zeng, Zhengkun Li, Zhonghua Zhang, Jiubin Tan

**Affiliations:** 1 School of Electrical Engineering and Automation, Harbin Institute of Technology, Harbin 150080, China; baiyang@hit.edu.cn (Y.B.); hupc@hit.edu.cn (P.H.); zengtao806@sina.com (T.Z.); jbtan@hit.edu.cn (J.T.); 2 National Institute of Metrology, Beijing 100013, China; lzk@nim.ac.cn (Z.L.); zzh@nim.ac.cn (Z.Z.); 3 Key Laboratory for the Electrical Quantum Standard of AQSIQ, Beijing 100029, China; 4 School of Instrumentation Science and Opto-Electronics Engineering, Beihang University, Beijing 100191, China; wywanggang163@163.com; 5 Department of Electrical Engineering, Tsinghua University, Beijing 100084, China; xujinxinthu@126.com

**Keywords:** capacitive sensor, length metrology, fringe effect

## Abstract

A simple differential capacitive sensor is provided in this paper to measure the absolute positions of length measuring systems. By utilizing a shield window inside the differential capacitor, the measurement range and linearity range of the sensor can reach several millimeters. What is more interesting is that this differential capacitive sensor is only sensitive to one translational degree of freedom (DOF) movement, and immune to the vibration along the other two translational DOFs. In the experiment, we used a novel circuit based on an AC capacitance bridge to directly measure the differential capacitance value. The experimental result shows that this differential capacitive sensor has a sensitivity of 2 × 10^−4^ pF/μm with 0.08 μm resolution. The measurement range of this differential capacitive sensor is 6 mm, and the linearity error are less than 0.01% over the whole absolute position measurement range.

## 1. Introduction

Capacitive sensors are widely used within the field of high precision geometrical measurements, including length and angle measurement [1,2,3,4]. Their advantages mainly lie in their simple structure, non-contact measurement and absolute position measurement capability. However, for high-precision length metrology systems, the measurement range of capacitive sensors is usually not larger than 1 mm, and the linearity interval of the capacitor sensor is quite limited because of the stray capacitances and fringe effect [5,6]. In comparison, laser interferometers or electrical encoders have longer measurement ranges and nanometer measurement accuracy. However, they are high-cost and cannot measure the absolute position in most cases [7,8].

Compared with single-ended capacitive sensors [9], differential capacitive sensors have the advantages of larger measurement range and higher sensitivity. Normally, the basic principle of this sensor is to change the effective overlap areas by moving one of the capacitive plates. Lots of research has been done to develop different types of differential capacitive sensors. For example, Ahn [10] measured the target position values by changing the overlap area of two cylindrical sensor electrodes, which had a linearity range about 10 mm; Ferri [11] used a differential capacitive sensor with a common electrode to respond to stimuli (such as displacements), whose nonlinearity error was less than 1.8%; Lee [12] presented a differential capacitive position sensor based on a microelectromechanical system (MEMS) to develop a sub-nanometer linear and micro-degree rotational position resolution. However, it should be noted that the sensor is easily disturbed when the capacitive plates move. For instance, it is hard to maintain a fixed gap between the plates during the movement of plates. This disturbance may give rise to noise during the capacitance measurement and harm the sensor linearity.

In this paper, a simple differential capacitive sensor is provided to measure absolute positions in length measurement systems. In this sensor, the capacitive plates do not move relative to each other, and instead, a grounded shield window moves inside the differential capacitive sensor to change the effective overlap areas of the capacitor. This sensor has a larger measurement range than normal capacitive sensors [13,14], and shows a linear relationship through the whole measurement range. What is more interesting is that this differential capacitive sensor is only sensitive to one translational DOF movement, and immune to vibrations along the other two translational DOFs. With a novel circuit based on an AC capacitance bridge, the differential capacitance value can be measured directly. The experimental results show that this differential capacitive sensor has a sensitivity of 2 × 10^−4^ pF/μm with 0.08 μm resolution. The measurement range of this differential capacitive sensor is 6 mm, and the linearity error are less than 0.01% over the whole absolute position measurement range.

## 2. Design of the Differential Capacitive Sensor

**Figure 1:**
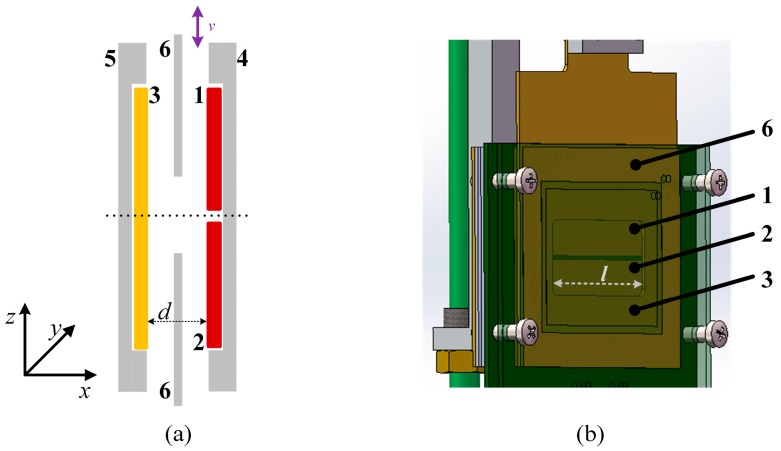
Schematic of differential capacitive sensor. (**a**) 2D schematic of differential capacitive sensor; (**b**) 3D schematic of differential capacitive sensor.

A schematic of the differential capacitive sensor is shown in Figure 1. Plate 1 and 2 are high voltage plates and Plate 3 is a low voltage plate, so two sub-capacitors are formed between Plates 1, 3 (*C*_1_) and Plates 2, 3 (*C*_2_). Plates 4 and 5 are grounded shields reducing the stray capacitances from the environment. Plate 6 is a grounded shield window, which moves back and forward along the z-axis.

It is easy to understand that the number of electrical potential lines between the plates of sub-capacitors are related to the position of the shield window along the z-axis. Regardless of the fringe effect, the relationship between the differential capacitance value and displacement of shield window is expressed as:

(1) $$\Delta C=\frac{2\varepsilon \cdot \varepsilon_{0}\left(l\cdot \Delta L\right)}{d}$$

where Δ*C* is the differential capacitance value between *C*_1_ and *C*_2_; ε is the relative permittivity; ε_0_ is the vacuum permittivity; *d* is the distance between the high voltage capacitor plate and low voltage capacitor plates; *l* denotes the width of the window. Δ*L* is the displacement away from the reference position. We define the position where Δ*C* = 0 is the measurement reference position.

**Figure 2:**
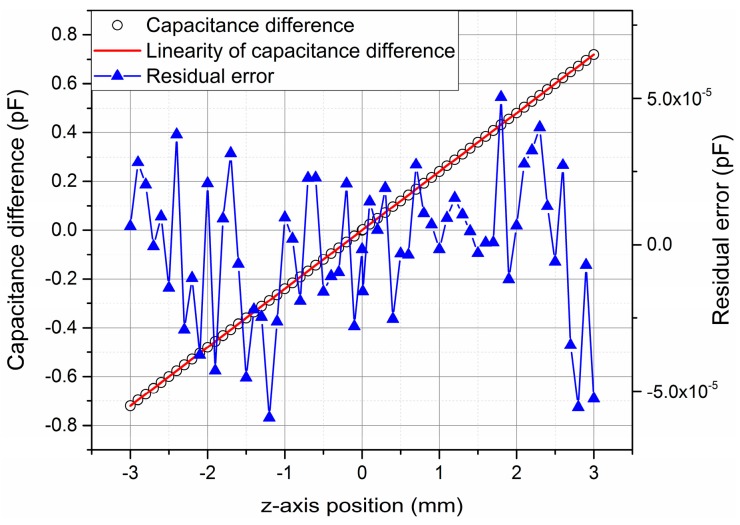
Simulation result of differential capacitance value over the range of ±3 mm along the z-axis.

In order to simulate or experimentally verify the performance of the sensor, a 3D model was built up with the dimensions *l* = 40 mm, *d* = 3 mm. According to Equation (1), the sensitivity of the differential capacitive sensor is 2.36 × 10^−4^ pF/μm along the z-axis and it shows a linear relationship with Δ*L*. Taking the fringe effect of the capacitors into account, we simulate the sensitivity and linearity of the sensor along the z-axis with the electrical finite element analysis method. The simulation result is shown in Figure 2. The sensitivity of the differential capacitive sensor is 2.4 × 10^−4^ pF/μm along the z-axis, and the residual errors between the differential capacitance values and the linearity values are within ±5 × 10^−5^ pF, that correspond to residual errors within about ±0.2 μm over the 6 mm measurement range.

**Figure 3:**
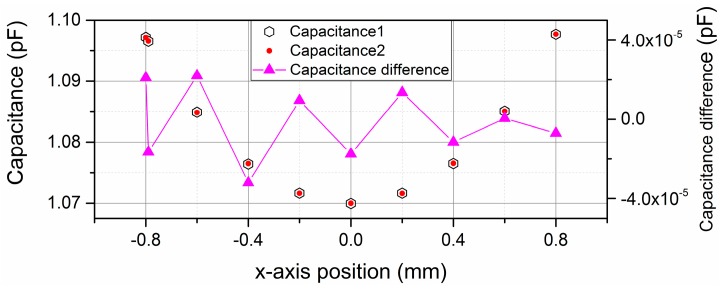
Simulation result of differential capacitance value over the range of ±0.8 mm along the x-axis.

As to the position variation in the x-axis, the movement direction of the shield window is parallel to the electric field lines, so this movement does not affect the effective overlap area of the differential capacitor, and only affects the fringe effect of the sensor. We define the mid-position between the high and low voltage plates along the x-axis as the zero position, and the simulation result over the measurement range from −0.8 mm to 0.8 mm is shown in Figure 3.

When the shield window moves from the −0.8 mm to 0.8 mm along the x-axis, the variation of the differential capacitance value is not larger than ±4 × 10^−5^ pF, so the absolute position measurement uncertainty caused by x-axis position disturbance is no larger than ±0.17 μm within the range of ±0.8 mm.

**Figure 4:**
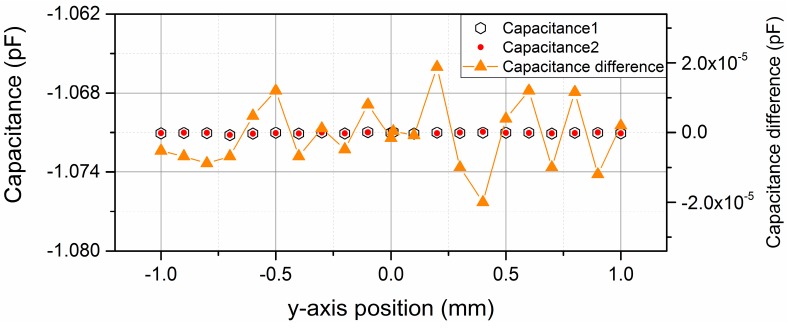
Simulation result of differential capacitance value over the range of ±1 mm along the y-axis.

As to the position variation in the y-axis, neither the effective areas nor the fringe effect of the two sub-capacitors changes, so theoretically the measurement uncertainty caused by y-axis position disturbances can be ignored. The simulation results over the ±1 mm measurement range along the y-axis are also presented in Figure 4. The symmetric position along the y-axis is defined as the reference position. When the shield window moves from the −1 mm to 1 mm along the y-axis, the variation of the differential capacitance value is no larger than ±2 × 10^−5^ pF, so the absolute position measurement uncertainty caused by y-axis position disturbance is no larger than ±0.08 μm within the range of ±1 mm.

## 3. Experimental Setup and Results

**Figure 5:**
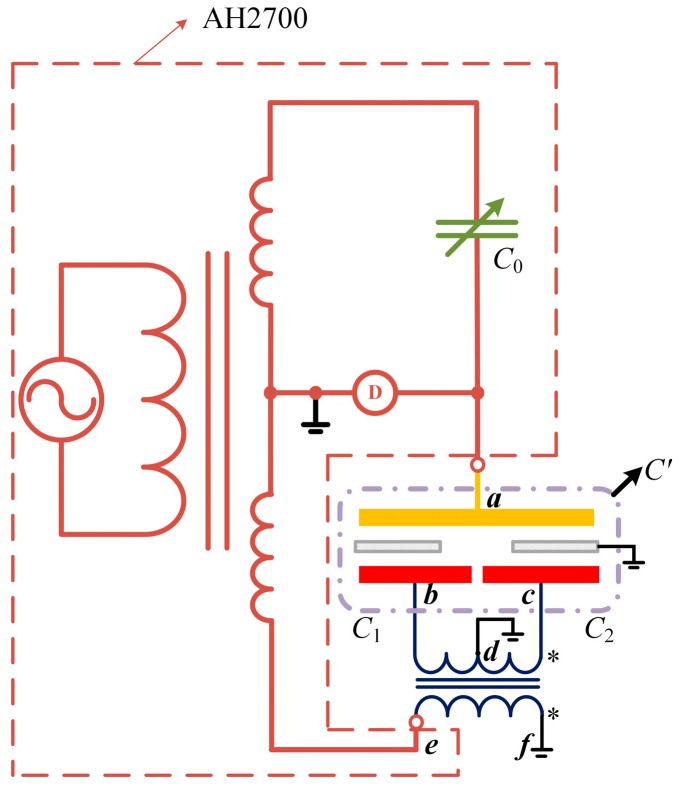
Schematic of the differential capacitance measurement circuit.

A novel circuit is designed and built based on an AC capacitance bridge (AH2700H) to directly measure the differential capacitance value [15,16]. The schematic of the circuit is shown in Figure 5. 

*C*_1_ and *C*_2_ are two sub-capacitors in this sensor; *C*_0_ is an adjustable standard capacitor in the AH2700A (marked in a dotted box); D in a circle denotes the zero indicator inside the AH2700A. When the AC capacitance bridge is balanced, the potential of point **a** is zero. The turns ratio of the two coils between the point **e**, **f** and **b**, **c** is 1:1 and the point **d** is the central tap of the **b**, **c** coil. We connect point **d** to the ground. If the potential of **e** is $\overset{•}{U}$, then:

(2) $$\left\{ \begin{array}{l} {\overset{•}{U}}_{b}=1/2\overset{•}{U}\\ {\overset{•}{U}}_{c}=-1/2\overset{•}{U} \end{array}\right.$$

the currents running through the two sub-capacitors are:

(3) $$\left\{ \begin{array}{l} {\overset{•}{I}}_{ab}=\frac{-1/2\overset{•}{U}}{1/j\omega C_{1}}=-j\omega \overset{•}{U}\frac{C_{1}}{2}\\ {\overset{•}{I}}_{ac}=\frac{1/2\overset{•}{U}}{1/j\omega C_{2}}=j\omega \overset{•}{U}\frac{C_{2}}{2} \end{array}\right.$$

so the total current between **a** and **e** is:

(4) $${\overset{•}{I}}_{ae}={\overset{•}{I}}_{ab}+{\overset{•}{I}}_{ac}=j\omega \overset{•}{U}\cdot \frac{1}{2}(C_{2}-C_{1})$$

**Figure 6:**
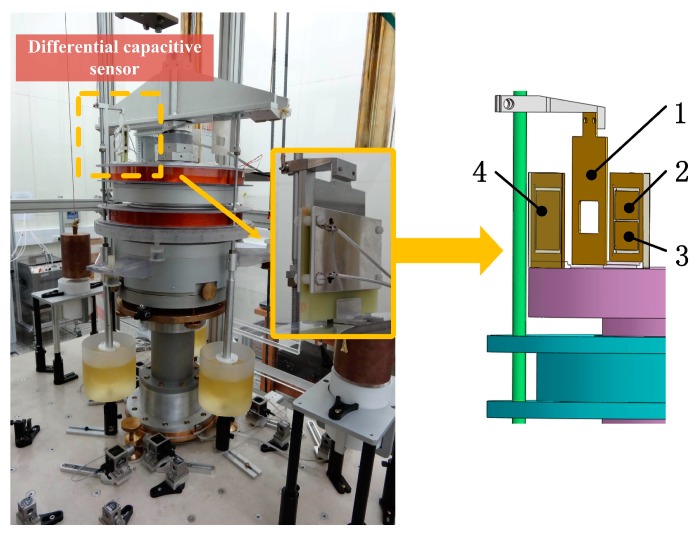
Experimental setup in Joule balance. **1**. Grounded shield window; **2**, **3**. High-voltage plates; **4**. Low-voltage plate.

Therefore the equivalent capacitor between **a** and ***e*** is $C^{\prime }=1/2(C_{2}-C_{1})$. We used this differential capacitive sensor to measure the relative position between the exciting coil and suspended coil in a Joule balance. The Joule balance is one of the apparatus used to redefine the kilogram, and the absolute position between the two coils has to be measured for an accurate nonlinearity compensation of the magnet assembly [17,18]. The differential capacitive sensor we design is employed here to measure the absolute position between the two coils. The shield window was mounted on the suspended coil and the capacitor plates were mounted on the exciting coil, giving the measurement system shown in Figure 6. The plates of the capacitance sensor were made from print circuit board, whose surfaces were plated with gold, and the shield windows are made of aluminum alloy.

Firstly, we align the differential capacitive sensor before any measurements. As to the parallelism between the high voltage plates and the low voltage plate, we use four equal-thickness nylon heel blocks mount around the four corners of the sensor, so that the parallelism between the high voltage plate and low voltage plates can be guaranteed, as shown in Figure 1b. Afterwards, we adjust the parallelism between the capacitor plates and moving direction until the sensitivity is the largest. Then, we adjust the angle between the shield window and the capacitive plates until the sub-capacitor values are the largest.

**Figure 7:**
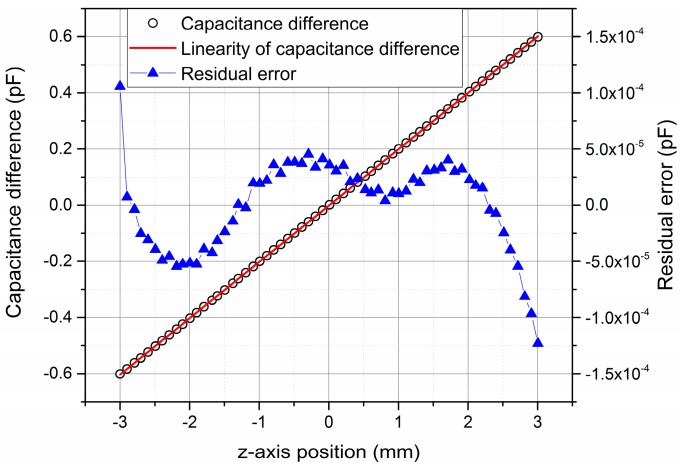
Calibrating linearity of differential capacitive sensor with laser interferometer.

When the sensor was in an aligned state, the linearity of this differential capacitive sensor was experimentally verified and calibrated online. In this progress, we used a laser interferometer (Keysight 5530, Keysight, Santa Rosa, CA, USA) and the provided differential capacitive sensor to measure the relative position change between the two coils at the same time [19]. The experiment result is shown in Figure 7. The displacement values in the x-axis of this figure were read from the laser interferometer. It can be seen from the experimental result that the measured sensitivity of this differential capacitive sensor is 2.0 × 10^−4^ pF/μm, which is close to the simulation result 2.4 × 10^−4^ pF/μm, but not exactly the same. This is because the distance between the high voltage plate and the low voltage plates is not exactly 3 mm, but 3.3 mm (the thickness of the heel blocks is 3.3 mm). As to the linearity error, the value differences between the linearity result and the measured capacitance value are within ±1.2 × 10^−4^ pF in the full range, which corresponds to ±0.6 μm measurement uncertainty. That means the linearity error of this differential capacitive sensor is less than 0.01% through the whole measurement range. The experiment result is consistent with the theoretical analysis above.

**Figure 8:**
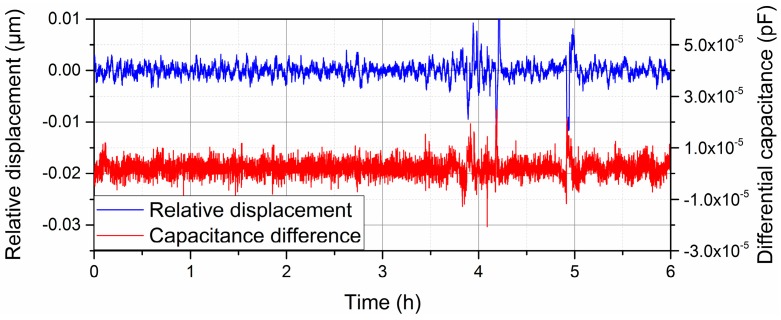
Long-time stability of the differential capacitance value under the position locking statement.

Then, we measured the stability of this differential capacitive sensor. The laser interferometer was also utilized to measure the relative displacement between the two coils and verify the performance of the differential capacitor sensor. In order to maintain a fixed relative position of the two coils along z-axis, a piezoelectric ceramic (PZT) and feedback control system are utilized to compensate the vibration and drift of relative displacement between the two coils [20]. The experiment result is shown in Figure 8. In this progress, we used an air sensor (Keysight E1736A, Keysight, Santa Rosa, CA, USA) to compensate the air index variation in the laser interferometer system.

When the short duration position vibration is within 10 nm, the short duration vibration of the differential capacitance value is within 1.5 × 10^−5^ pF, so the resolution of this sensor is 1.5 × 10^−5^/2.0 × 10^−4^ = 0.08 μm. It should be noticed that the bandwidth of this measuring system is no larger than 26 Hz, because of the capacitance bridge method. The measurement speed is limited according to the operation and maintenance manual of AH2700A [21]. For a long duration experiment, the capacitance changes due to the alterations in the permittivity of the air should also be considered, which are closely related to the temperature, pressure, and relative humidity of the air. In our lab the variation ranges of air temperature, pressure, and relative humidity are 19.8 ± 0.1 °C, 100 ± 0.4 kPa, 48% ± 2%, respectively, during the measurement, so the capacitance value changes caused by the environment can be ignored in this experiment [22,23,24].

**Figure 9:**
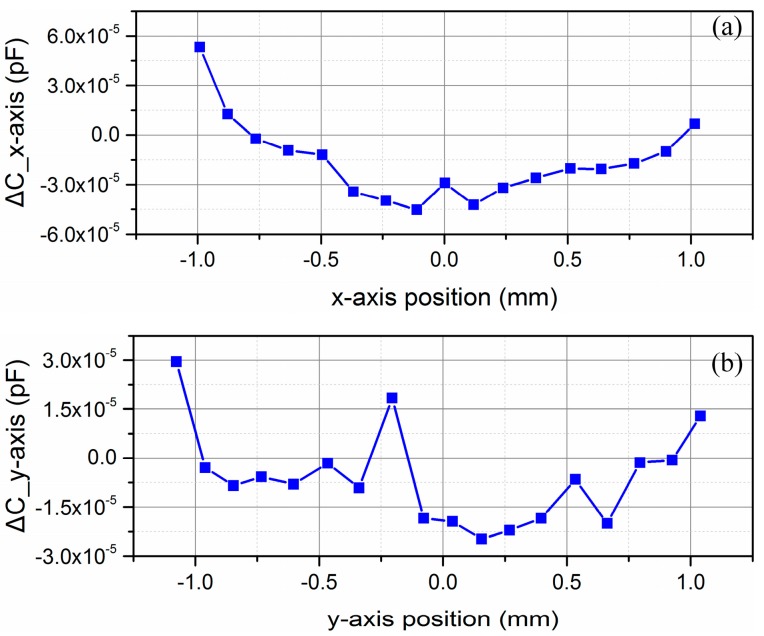
Experimental results of differential capacitance value along x and y axes. (**a**) Experimental result over the range of ±1 mm along the x-axis; (**b**) Experimental result over the range of ±1 mm along the y-axis.

As to the position vibration along x and y axes, the sensitivity of this differential capacitive sensor have also been experimentally tested. In this experiment, another linear guide (MTMS103) were utilized to relatively change the positon between the shield window and the capacitive plates along the x and y axes, respectively. At the same time, a laser interferometer (SP 2000, SIOS, Ilmenau, Germany) measured the position variation, and a capacitance bridge system described above measured the differential capacitance value. The experimental result is shown in Figure 9a,b.

From Figure 9a, when the position variation along x-axis is within ±1 mm in the experiment, the variation of differential capacitive value is within ±5 × 10^−5^ pF, so the position measurement uncertainty caused by x-axis disturbance is no larger than ±0.25 μm within the range of ±1 mm. As to the position variation along the y-axis in Figure 9b, the differential capacitive value is within ±3 × 10^−5^ pF, so the position measurement uncertainty caused by y-axis disturbance is no larger than ±0.15 μm within the range of ±1 mm.

Taking the aforementioned factors into account, the measurement uncertainty of this differential capacitive sensor is shown in Table 1. Because of the online calibration, the measurement uncertainty caused by alignment errors can be eliminated. Therefore from Table 1, the combined measurement uncertainty of this differential capacitor sensor is 0.67 μm in the full measurement range. The largest uncertainty comes from the residual errors of the measurement value and linearity value. From Figure 7, most of the residual errors are actually within ±5 × 10^−5^, which corresponds to 0.25 μm measurement uncertainty, so if the measurement range of this differential capacitive sensor could become slightly shorter, like ±2.5 mm, the combined measurement uncertainty of this sensor will decline to 0.39 μm.

Compared with former research works, the main feature of the proposed sensor lies in that we do not relatively move the capacitive plates to change the overlap areas. Instead, we use a shield window to move inside the differential capacitive sensor to change the areas, so better linearity, measurement range and immunity to disturbances can be achieved. Though this method looks similar to the method of altering the dielectric constant of the insulating layer [25], the main difference is that the dielectric of the proposed differential capacitive sensor is always air.

## 4. Conclusions

In this article, we focus on describing a novel but simple differential capacitive sensor to measure the absolute positions of length measurement systems. In this sensor, a shield window moves relative to the capacitor plates along the z-axis, so that the measurement range and linearity range can become much larger than possible with normal differential sensors, and the sensor has a linear output through the whole measurement range. In order to verify the feasibility of this sensor, we have simulated and experimentally tested a specified size sensor. The experimental results show that this differential capacitive sensor has a sensitivity of 2 × 10^−4^ pF/μm with 0.08 μm resolution. The measurement range of this differential capacitive sensor is 6 mm, and the linearity error is less than 0.01% over the whole absolute position measurement range. This result is consistent with the theoretical analysis. In addition, this sensor is only sensitive to one translational DOF movement, and immune to the vibration along the other two translational DOFs. We believe the sensor proposed in this paper can contribute to large-scale absolute position measurement, and it should be easy to expand for a larger measurement range and higher sensitivity for different applications.

## Figures and Tables

**Table 1:** Measurement uncertainty of differential capacitive sensor.

Error Sources	Uncertainty (μm)
Residual error between the measurement value and linearity value	0.6
Movement along x-axis	0.25
Movement along y-axis	0.15
Short-time vibration	0.08
Combined	0.67
